# RNA Sequencing Provides Insights into the Regulation of Solanesol Biosynthesis in *Nicotiana tabacum* Induced by Moderately High Temperature

**DOI:** 10.3390/biom8040165

**Published:** 2018-12-07

**Authors:** Ning Yan, Yongmei Du, Hongbo Zhang, Zhongfeng Zhang, Xinmin Liu, John Shi, Yanhua Liu

**Affiliations:** 1 Tobacco Research Institute of Chinese Academy of Agricultural Sciences, Qingdao 266101, China; duyongmei@caas.cn (Y.D.); zhanghongbo@caas.cn (H.Z.); zhangzhongfeng@caas.cn (Z.Z.); liuxinmin@caas.cn (X.L.); 2 Guelph Food Research Center, Agriculture and Agri-Food Canada, Guelph, ON N1G 5C9, Canada; john.shi@agr.gc.ca

**Keywords:** *Nicotiana tabacum*, solanesol, moderately high temperature, RNA-sequencing, functional enrichment analysis, gene expression

## Abstract

Solanesol is a terpene alcohol composed of nine isoprene units that mainly accumulates in solanaceous plants, especially tobacco (*Nicotiana tabacum*). The present study aimed to investigate the regulation of solanesol accumulation in tobacco leaves induced by moderately high temperature (MHT). Exposure to MHT resulted in a significant increase in solanesol content, dry weight, and net photosynthetic rate in tobacco leaves. In MHT-exposed tobacco leaves, 492 and 1440 genes were significantly up- and downregulated, respectively, as revealed by RNA-sequencing. Functional enrichment analysis revealed that most of the differentially expressed genes (DEGs) were mainly related to secondary metabolite biosynthesis, metabolic pathway, carbohydrate metabolism, lipid metabolism, hydrolase activity, catalytic activity, and oxidation-reduction process. Moreover, 122 transcription factors of DEGs were divided into 22 families. Significant upregulation of *N. tabacum* 3-hydroxy-3-methylglutaryl-CoA reductase (*NtHMGR*), 1-deoxy-d-xylulose 5-phosphate reductoisomerase (*NtDXR*), geranylgeranyl diphosphate synthase (*NtGGPS*), and solanesyl diphosphate synthase (*NtSPS*) and significant downregulation of *N. tabacum* 1-deoxy-d-xylulose 5-phosphate synthase (*NtDXS*) and farnesyl diphosphate synthase (*NtFPS*) transcription upon MHT exposure were monitored by quantitative real-time PCR (qRT-PCR). This study indicated that solanesol accumulation in tobacco leaves can be manipulated through regulation of the environmental temperature and established a basis for further elucidation of the molecular mechanism of temperature regulation of solanesol accumulation.

## 1. Introduction

In China, tobacco (*Nicotiana tabacum*) is mainly used as an industrial raw material for cigarette production; however, with the increasing stringent measures against tobacco in China, the development of tobacco planting industry has been limited [1]. However, a variety of valuable phytochemicals (such as solanesol and cembranoids) extracted from tobacco can be used in food, medicine, and other industries; these phytochemicals can provide effective ways to expand available resources and seek new drug sources [2,3]. Solanesol, which mainly accumulates in solanaceous plants, especially tobacco, is a noncyclic terpene alcohol comprised of nine isoprene units [2,4,5,6]. Solanesol has antibacterial, antifungal, antiviral, anticancer, anti-inflammatory, and anti-ulcer activities [2]. Moreover, it is an important drug intermediate that can be used to synthesize Coenzyme Q_10_ (CoQ_10_), vitamin K_2_, and other ubiquinone drugs, and the anticancer potentiator N-solanesyl-N,N′-bis(3,4-dimethoxybenzyl) ethylenediamine (SDB) [2,4,6]. As solanesol derivatives, CoQ_10_ is used to treat cardiovascular disease, migraine, Parkinson’s disease, and neurodegenerative diseases (e.g., Huntington disease), and as a dietary supplement for patients with type 2 diabetes; vitamin K_2_ has been used to treat osteoporosis, and SDB can overcome P-glycoprotein-mediated multidrug resistance of cancer cells [2,6,7]. Recently, Yao et al. [8,9] have found that solanesol not only can induce HO-1 and Hsp70 expression, thereby mitigating alcohol-induced hepatocyte injury, but it also displays anti-inflammatory activity by inhibiting the production of inflammatory cytokines through the p38 and Akt signaling pathways. Moreover, Qin et al. [10,11] have reported that mPEGylated solanesol micelles increase the oral bioavailability of CoQ_10_ and serve as redox-responsive nanocarriers with synergistic anticancer effect.

Chemical synthesis of solanesol is difficult [12]; therefore, it is primarily extracted from solanaceous plants, particularly, from tobacco leaves [2,4]. Solanesol accumulation in plants is affected by genetic and environmental factors [2,6,13]. Solanesol content varies widely among tobacco varieties [2,14]. To identify tobacco stocks with high solanesol content, we had determined the solanesol content of 168 Chinese flue-cured tobacco germplasm resources planted in four geographical regions of China in 2014 and 2015; we found that their contents were within the range of 0.70–4.13% [14]. The ‘major gene plus polygene’ mixed inheritance model showed that solanesol content in tobacco leaves is controlled by two pairs of ‘co-dominant major gene plus additive dominant polygene’, with major gene transmission rates of 33.61% and 53.15% and polygene transmission rates of 30.01% and 13.64% [15]. After infection with tobacco mosaic virus (TMV) and *Pseudomonas syringae* pv. *tabaci*, resistant varieties showed significantly higher solanesol content, while susceptible varieties exhibited no significant increases in solanesol content, indicating that solanesol plays an important role in the response of tobacco to biotic stress [16]. Previous studies have shown that drought, shade, long-wavelength light, and rare earth element treatments all lead to increased solanesol content [2,13]. However, there have been few studies on the influence of environmental temperature on solanesol accumulation in tobacco leaves.

Transcriptome analysis has been successfully used to study the molecular mechanisms of plant responses to environmental changes [17,18,19]. In a broad sense, the transcriptome refers to the collection of all RNAs—including messenger RNA (mRNA), ribosomal RNA, transfer RNA, and non-coding RNA—transcribed by a cell or tissue of an organism in a specific state; in a narrow sense, it refers to the collection of all mRNAs [20]. Oligo(dT) magnetic beads can be used to enrich all mRNAs transcribed by a specific tissue or cell at any specific time or location that can be sequenced using the Illumina HiSeq platform [21]. In this way, mRNA sequences and their abundances can be rapidly and comprehensively analyzed, and new genes and new transcripts can be identified [22]. Transcriptome sequencing can be used to identify differentially expressed genes (DEGs) related to different phenotypes under different treatment conditions [17,18,19]. Gene Ontology (GO) and Kyoto Encyclopedia of Genes and Genomes (KEGG) metabolic pathway predictions can be used for enrichment analysis and for categorizing DEGs [23]. The most active genes among DEGs can be identified to guide further research and assist in elucidating biological mechanisms [24].

Solanesol biosynthesis occurs in chloroplasts of higher plants via the 2-*C*-methyl-d-erythritol 4-phosphate (MEP) pathway [2,5,6]. Moreover, chloroplasts are organelles for plant photosynthesis, that use chlorophyll to convert light energy into chemical energy, transforming CO_2_ and water into sugar [25]. In our previous study, 30 candidate genes involved in solanesol biosynthesis were identified, including 1-deoxy-d-xylulose 5-phosphate synthase (*DXS*), 1-deoxy-d-xylulose 5-phosphate reductoisomerase (*DXR*), 2-*C*-methyl-d-erythritol 4-phosphate cytidylyltransferase (*IspD*), 4-diphosphocytidyl-2-*C*-methyl-d-erythritol kinase (*IspE*), 2-*C*-methyl-d-erythritol 2,4-cyclo-diphosphate synthase (*IspF*), 1-hydroxy-2-methyl-2-(*E*)-butenyl 4-diphosphate synthase (*IspG*), 1-hydroxy-2-methyl-2-(*E*)-butenyl 4-diphosphate reductase (*IspH*), isopentenyl diphosphate isomerase (*IPI*), and solanesyl diphosphate synthase (*SPS*) genes [5]. In *Arabidopsis thaliana*, *AtSPS1* overexpression lightened the damage induced by photooxidative stress, and *AtSPS1* and *AtSPS2* gene silencing induced photosystem II photoinhibition [26,27]. The objective of the present study was to evaluate the effects of moderately high temperature (MHT) on solanesol accumulation in tobacco leaves and to determine the underlying regulatory mechanisms, by comparing solanesol contents, and net photosynthetic rate, and through RNA-sequencing-based transcriptome analysis of tobacco leaves under normal temperature (NT) and MHT conditions. This study indicates that solanesol accumulation in tobacco leaves can be manipulated through the regulation of the environmental temperature and revealed the effect of MHT on the transcription of genes essential to solanesol biosynthesis, thus establishing a basis for further elucidation of the molecular mechanism of temperature regulation of solanesol accumulation.

## 2. Materials and Methods

### 2.1. Plant Materials and Growing Conditions

Tobacco (*N. tabacum* ‘Honghuadajinyuan’) seeds were obtained from the China Tobacco Germplasm Platform (www.ycsjk.com.cn, Qingdao, China). The seeds were germinated in growing medium containing a mixture of peat and vermiculite (2:1, v/v) in a greenhouse. Following germination, plants (~12 cm high) were transferred to pots (12-cm diameter, one plant per pot) containing the above-described growing medium. After eight weeks, the plants were subdivided into two sets and were moved to an illuminated incubator under conditions of either NT (day/night temperature, 22 °C/16 °C) or MHT (day/night temperature, 30 °C/24 °C). Other environmental conditions were the same for the two treatment groups: a 12 h photoperiod and a photosynthetic photon flux density of 800 μmol·m^–2^·s^1^. Plants were watered every two days with Hoagland’s nutrient solution (sourced from China Scientific Research Material Purchasing Platform: https://www.caasbuy.com/). Leaf samples from biological triplicates were harvested from the third fully expanded leaf from the apex of the plant at 0, 3, 6, 9, and 12 days after treatment (DAT).

### 2.2. Analysis of Total Solanesol Content

Tobacco leaves were dried to constant weight using a freeze-dryer (Alpha 1–2 LD Plus; Christ, Osterode am Harz, Germany), ground, and sifted through a 40-mesh sieve. Portions (0.2 g) of the powdered samples were transferred to individual 20-mL centrifuge tubes with stoppers, and 1 mL 1 M NaOH (diluted in ethanol) and 5 mL hexane was added. Ultrasonic extraction was performed at 50 °C for 30 min, and 8 mL distilled water was added. After centrifugation at 3000× *g* for 10 min, 0.5 mL of the supernatants was taken, diluted with 4.5 mL methanol in brown volumetric flasks, and filtered through a 0.2 μm membrane. Total solanesol contents were measured using ultra-high performance liquid chromatography (ACQUITY UPLC H-Class; Waters, Milford, MA, USA) with an Atlantis T3-C_18_ column (4.6 × 150 mm, 3 μm; Waters) that was maintained at 35 °C. A 50:50 (v/v) methanol-acetonitrile solution was used as the mobile phase at a flow rate of 1.0 mL/min, and a diode array detector was used for detection at 208 nm [5].

### 2.3. Measurement of Leaf Dry Weight and Net Photosynthetic Rate

Leaf dry weight was measured from the third fully expanded leaf from the apex of the plant at 0, 3, 6, 9, and 12 DAT. The tobacco leaves were dried to constant weight with an oven before the measurement of dry weight. Net photosynthetic rate was measured with a LI-6400 portable photosynthesis system (Li-Cor, Lincoln, Dearborn, MI, USA) at 25 °C under artificial light of 1200 μmol·m^−2^·s^−1^ and CO_2_ concentration of 350 μL·L^−1^.The third fully-expanded leaf from the top of a plant was measured at 0, 3, 6, 9, and 12 DAT. The measurements were performed according to the method of Yan et al. [28,29].

### 2.4. RNA Extraction and Sequencing (RNA-Seq)

Total RNA was extracted from tobacco leaves grown at NT and MHT from 9 DAT following the protocol of Wang et al. [19] using TRIzol^®^ (Thermo Fisher Scientific, Carlsbad, CA, USA). RNA integrity was assessed using the RNA Nano 6000 Assay Kit of the Bioanalyzer 2100 system (Agilent Technologies, Santa Clara, CA, USA). RNA-sequencing of two samples, namely NT and MHT, was performed using Illumina HiSeq platform (Illumina Co., San Diego, CA, USA), and 125-bp/150-bp paired-end reads were generated. The RNA extraction and sequencing experiments were repeated three times.

### 2.5. RNA-Seq Quality Control and Reads Mapping to the Reference Genome

Raw read data in FASTQ format were first processed using in-house Perl scripts. Reads containing adapter, reads containing poly-N, and low-quality reads were removed. Q20 (corresponds to the base calling accuracy of 99%), Q30 (corresponds to the base calling accuracy of 99.9%), and GC content of the clean data were calculated. Reference genome and gene modelannotation files were downloaded from ftp://ftp.ncbi.nlm.nih.gov/genomes/all/GCF/000/715/135/GCF_000715135.1_Ntab-TN90 [30]. The reference genome was indexed using Bowtie v2.2.3 [31], and the clean reads were aligned to the reference genome using TopHat v2.0.12 [32].

### 2.6. Quantification of Gene Expression Levels and Differential Gene Expression Analysis

The fragments per kilobase of exon per million fragments mapped (FPKM) of each gene was calculated using HTSeq v0.6.1 [33]. FPKM considers sequencing depth as well as gene length for the read count and is currently the most commonly used method for estimating gene expression levels [22]. DEGs of NT and MHT were identified using the DESeq R package (1.18.0). DESeq provide statistical routines for determining differential expression in digital gene expression data using a model based on the negative binomial distribution [34]. The obtained *p*-values were adjusted using the Benjamini and Hochberg’s approach for controlling the false discovery rate. In the present study, genes with an adjusted *p*-value (padj) of <0.05 were considered differentially expressed.

### 2.7. GO and KEGG Enrichment Analysis of Differentially Expressed Genes

Gene ontology enrichment analysis of DEGs was conducted using the GOseq R package, in which gene length bias was corrected [35]. In the present study, GO terms with corrected *p* < 0.05 were considered significantly enriched by DEGs. Moreover, we used KOBAS 2.0 software to test the statistical enrichment of DEGs in KEGG pathways [36].

### 2.8. Quantitative Real-Time PCR

The quantitative real-time PCR (qRT-PCR) was used to monitor the transcription of six key enzyme genes involved in solanesol biosynthesis. RNA was extracted from samples of tobacco leaves collected at five stages (0, 3, 6, 9, and 12 DAT) using TRIzol^®^ reagent (Takara, Shiga, Japan). RNA was reverse-transcribed into complementary DNA (cDNA) using a PrimeScript^®^ RT reagent Kit (Takara). Reactions were run in triplicate on an Applied Biosystems 7500 Real Time PCR system (Thermo Fisher Scientific), in a final volume of 25 μL, containing 12.5 μL SYBR^®^ Premix Ex Taq II (2×), 1.0 μL forward and reverse primer (10 μM), 2.0 μL cDNA (50 ng/μL), and 8.5 μL ddH_2_O. Two-step PCR was performed according to the manufacturer’s procedure, with an initial denaturation step at 95 °C for 30 s, followed by 40 cycles of 95 °C for 5 s and 60 °C for 30 s. The actin gene was used as the internal control. Primers used for qRT-PCR are listed in Table 1.

### 2.9. Statistical Analysis

Data were analyzed by analysis of variance (ANOVA) using the SPSS package program version 19.0 (SPSS, Chicago, IL, USA). Data are reported as means ± standard deviation. Student’s *t*-test was used to determine significant differences between NT and MHT treatments. *p* < 0.05 was considered significant.

### 2.10. GenBank Accession Code

The sequence data generated for this work are accessible via the NCBI Sequence Read Archive under accession number (SRA: SRR7537985, SRR7537986, SRR7537983, SRR7537984, SRR7537987, and SRR7537988). 

## 3. Results

### 3.1. Total Solanesol Contents in Leaves of Tobacco Plants Grown at Normal Temperature and Moderately High Temperature 

**Figure 1:**
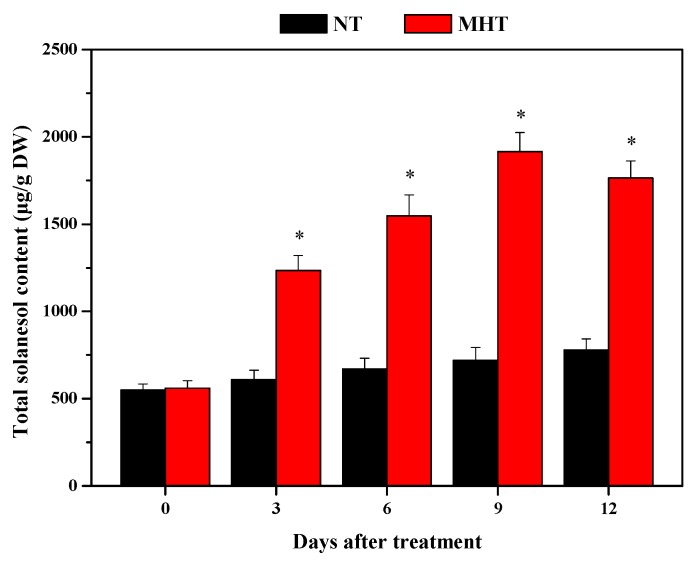
Total solanesol content of tobacco leaves grown at normal temperature (NT) and moderately high temperature (MHT). The asterisk (*) indicates *p* < 0.05. DW – dry weight.

At 0 DAT, there was no significant difference in total solanesol content of tobacco leaves grown at NT and those grown at MHT (*p* > 0.05). At 3, 6, 9, and 12 DAT, the leaf total solanesol contents of tobacco plants grown at MHT increased by 102.3%, 131.0%, 166.1%, and 126.2%, respectively, and were significantly higher (*p* < 0.05) than the concentrations in plants grown at NT (Figure 1). As expected, total solanesol content of tobacco leaves grown at NT slightly increased from 0 to 12 DAT (Figure 1).

### 3.2. Leaf Dry Weight and Net Photosynthetic Rate in Leaves of Tobacco Plants Grown at Normal Temperature and Moderately High Temperature

**Figure 2:**
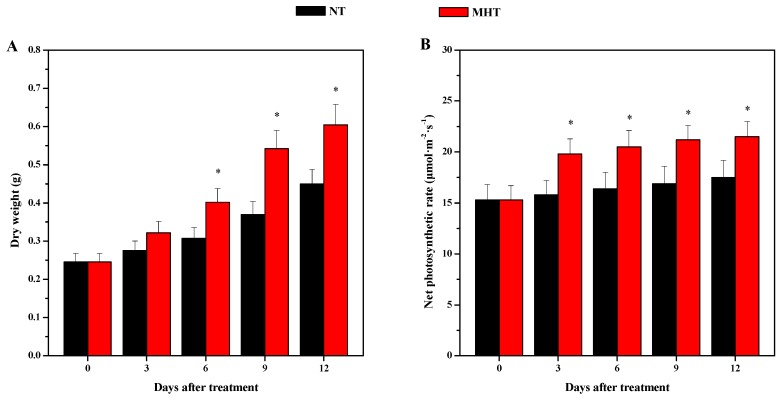
Dry weight (**A**) and net photosynthetic rate (**B**) of tobacco leaves grown at NT and MHT. The asterisk (*) indicates *p* < 0.05.

At 0 and 3 DAT, there was no significant difference in dry weight of tobacco leaves grown at NT and those grown at MHT (*p* > 0.05). At 6, 9, and 12 DAT, the leaf dry weight of tobacco plants grown at MHT increased by 30.5%, 46.5%, and 34.2%, respectively, and was significantly higher (*p* < 0.05) than that in plants grown at NT (Figure 2A). At 0 DAT, there was no significant difference in net photosynthetic rate of tobacco leaves grown at NT and those grown at MHT (*p* > 0.05). At 3, 6, 9, and 12 DAT, the leaf net photosynthetic rate of tobacco plants grown at MHT increased by 25.3%, 25.0%, 25.4%, and 22.9%, respectively, and was significantly higher (*p* < 0.05) than that in plants grown at NT (Figure 2B).

### 3.3. Quality Assessment of Sequencing Data, Sequence Alignment, and Transcription Analysis of Differentially Expressed Genes

#### 3.3.1. Quality Assessment of Sequencing Data

Information on sequencing data quality in this study is shown in Supplementary Table S1. Under normal conditions, the single-base sequencing error rate should be less than 1%. In this study, the sequencing error rates of NT_1, NT_2, NT_3, MHT_1, MHT_2, and MHT_3 were 0.01%, 0.01%, 0.01%, 0.01%, 0.02%, and 0.01%, respectively, which is far below 1%. Raw reads from both NT and MHT samples were processed to remove adaptor sequences and low-quality reads before data analysis. The numbers of clean bases in both samples were above 7.00 G. The distribution of GC content is examined to determine whether AT/GC segregation is present, which can result from sequencing or database construction and affect subsequent quantitative analyses. In this study, the GC content of NT_1, NT_2, NT_3, MHT_1, MHT_2, and MHT_3 was 43.88%, 43.50%, 43.66%, 43.67%, 43.64%, and 43.66%, respectively (Supplementary Table S1).

#### 3.3.2. Sequence Alignment

In the present study, whole read segments were mapped to genome exons, and exon-spanning read segments were aligned. Detailed statistics on mapping are shown in Supplementary Table S2. If the reference genome is selected appropriately and the samples are not contaminated, the percentage of total mapped reads will normally be over 70%, and multiple mapped reads account for no more than 10% of the total percentage. In this study, the percentages of total reads mapped to the reference genome for NT_1, NT_2, NT_3, MHT_1, MHT_2, and MHT_3 were 92.61%, 92.94%, 93.49%, 92.68%, 92.47%, and 93.01%, and the percentages of multiple mapped reads were 2.38%, 2.32%, 2.52%, 2.56%, 2.47%, and 2.42%, respectively (Supplementary Table S2).

The distribution of the reads mapped to exons, introns, and inter-genic regions was determined. In species with relatively complete genome annotations, the number of reads aligned to exons is the highest, reads aligned to intron regions originate from pre-mRNA residues and introns stranded during alternative splicing, and reads aligned to inter-genic regions are due to incomplete genome annotations. In this study, the reads for NT_1, NT_2, NT_3, MHT_1, MHT_2, and MHT_3 mapped to exon regions were 95.2%, 94.4%, 94.7%, 94.3%, 94.0%, and 94.1%, respectively (Supplementary Table S2). Thus, the reference genome in this study was selected appropriately, and the sequence alignment results are nearly ideal.

#### 3.3.3. Transcription Analysis of Differentially Expressed Genes

**Figure 3:**
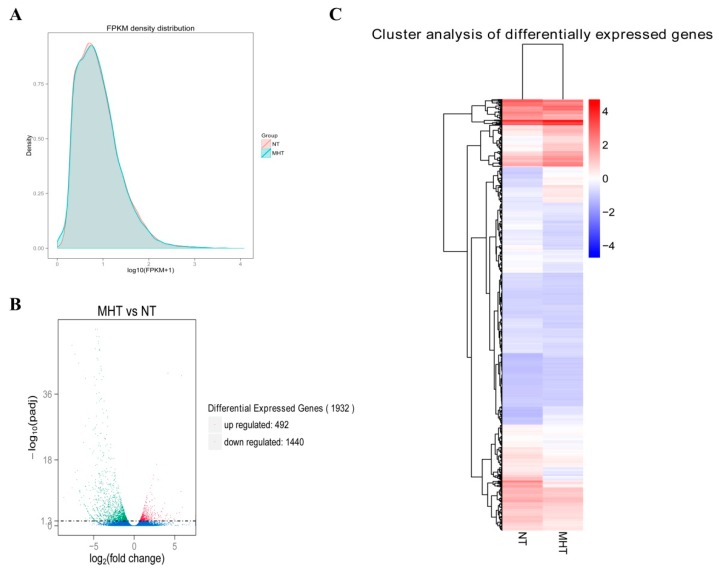
Expression analysis of differentially expressed genes (DEGs) between NT and MHT exposed tobacco leaves. (**A**) Fragments per kilobase of exon per million fragments mapped (FPKM) density distribution between NT and MHT tobacco leaf transcriptomes. (**B**) Volcano plot of DEGs between NT and MHT tobacco exposed leaves. The mean expression value of -log_10_ (padj) is plotted on the ordinate, and the log_2_ (fold change) value is plotted on the abscissa. Each dot represents an individual gene. Red dots represent significantly upregulated DEGs, and green dots represent significantly downregulated DEGs. Blue dots represent genes whose expression was not significantly different between the two treatment groups. (**C**) Hierarchical clustering analysis of DEGs between NT and MHT exposed tobacco leaves. The color scale indicates the log_10_ (FPKM + 1) values. Red indicates high gene expression, and blue indicates low gene expression.; Padj, adjusted *p*-value.

The fragments per kilobase of exon per million fragments mapped density distributions of NT and MHT tobacco leaf transcriptomes were compared. In Figure 3A, the log_10_ (FPKM) value of a gene is plotted on the abscissa and the density corresponding to the log_10_ (FPKM) value is plotted on the ordinate; the higher the value, the higher the gene expression level. As shown in Figure 3A, the log_10_ (FPKM) density distributions were slightly different between NT and MHT samples. A volcano plot depicting the relationship between the -log_10_ (padj) and log_2_ (fold change) is shown in Figure 3B. The log2 (fold change) in gene expression of NT and MHT exposed tobacco leaf transcriptomes is plotted on the abscissa. The degree of statistical significance of the changes in gene expression is plotted on the ordinate. As shown in Figure 3B; 492 and 1440 genes were significantly up- and downregulated, respectively, by MHT treatment as compared to NT treatment. A heat map of the FPKM values of the 1932 DEGs after clustering is shown in Figure 3C. Moreover, the 1932 DEGs were divided into 18 clusters by hierarchical clustering.

### 3.4. Gene Ontology Functional Enrichment Analysis of Differentially Expressed Genes

Gene ontology functional annotations of DEGs were compared between the treatment groups to identify biological functions that were significantly affected by MHT treatment [23,24]. In GO enrichment analysis based on the non-central hyper-geometric distribution [35], the probability of drawing an individual from within a certain group is different from the probability of drawing an individual from outside of that group. The results of GO functional enrichment analysis are shown in Table 2. Under ‘molecular function’ type, ‘hydrolase activity, hydrolysing *O*-glycosyl compounds’, ‘hydrolase activity, acting on glycosyl bonds’, ‘copper ion binding’, and ‘catalytic activity’ were significantly enriched in the plants exposed to MHT. Under ‘biological process’ type, ‘carbohydrate metabolic process’, ‘lipid metabolic process’, ‘oxidation-reduction process’, and ‘single-organism metabolic process’ were significantly enriched in the plants exposed to MHT. Thus, GO functional enrichment analysis indicated that MHT treatment affected genes related to the above processes.

### 3.5. Kyoto Encyclopedia of Genes and Genomes Functional Enrichment Analysis of Differentially Expressed Genes

**Figure 4:**
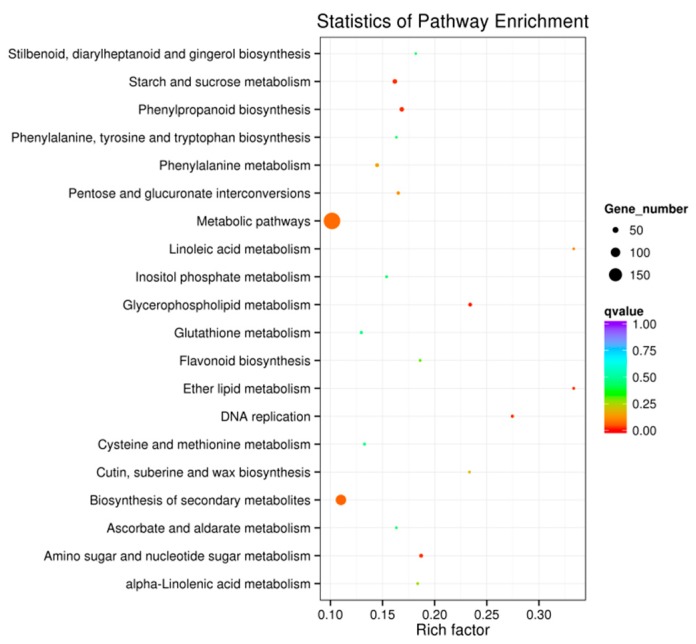
Statistics of Kyoto Encyclopedia of Genes and Genomes (KEGG) pathway enrichment analysis of DEGs between NT and MHT exposed tobacco leaves. KEGG pathways are plotted on the ordinate, and the enrichment factor (rich factor) is plotted on the abscissa. The color of points represents the q-value, and the size of points represents the number of DEGs mapped to the reference pathway. Legends for the color scale of q-values and size-scaling of the number of DEGs are shown to the right of the plot.

Kyoto Encyclopedia of Genes and Genomes pathway enrichment analysis was used to reveal the major metabolic and signal transduction pathways affected by MHT treatment [24,36]. The 20 most significantly enriched pathways in this study are shown in Figure 4. The q-value for ‘glycerophospholipid metabolism’ was <0.01, suggesting that genes in the related pathways were significantly enriched in MHT-exposed tobacco leaves. The q-values for ‘DNA replication’, ‘phenylpropanoid biosynthesis’, ‘ether lipid metabolism’, ‘starch and sucrose metabolism’, and ‘amino sugar and nucleotide sugar metabolism’ ranged between 0.01 and 0.05, suggesting that genes in these pathways were relatively enriched in MHT samples. The number of DEGs in the categories of ‘biosynthesis of secondary metabolites’ and ‘metabolic pathway’ were 114 and 199, respectively (Figure 4). Thus, KEGG functional enrichment analysis indicated that MHT treatment affected genes related to the biosynthesis of secondary metabolites and metabolic pathway.

### 3.6. Transcription Factor Analysis of DEGs

Transcription factors (TFs) are widely involved in plant abiotic stress responses [37]. In the present study, 122 TFs of DEGs were identified, as shown in Table 3. These TFs were divided into 22 families, including AP2-EREBP, bHLH, bZIP, C2C2-Dof, C2C2-GATA, C2H2, C3H, CCAAT, GRAS, HB, HSF, LIM, LOB, LUG, MADS, MYB, NAC, Orphans, PLATZ, S1Fa-like, SBP, SET, SNF2, TCP, TRAF, Trihelix, WRKY, and zf-HD. Among these families, the percentage of MYB (16, 13.11%), AP2-EREBP (12, 9.84%), NAC (12, 9.84%), bHLH (10, 8.2%), HB (9, 7.38%), and SBP (7, 5.74%) was more than 5% (Table 3). Notably, Orphans (4, 3.28%), SNF2 (2, 1.64%), TRAF (2, 1.64%), LUG (1, 0.82%), and SET (1, 0.82%) are transcriptional regulators. The FPKM values of 122 TFs of DEGs between NT and MHT exposed tobacco leaves were shown in Supplementary Table S3.

### 3.7. Transcription of Solanesol Biosynthesis Genes in Leaves of Tobacco Plants Grown at Normal Temperature and Moderately High Temperature

**Figure 5:**
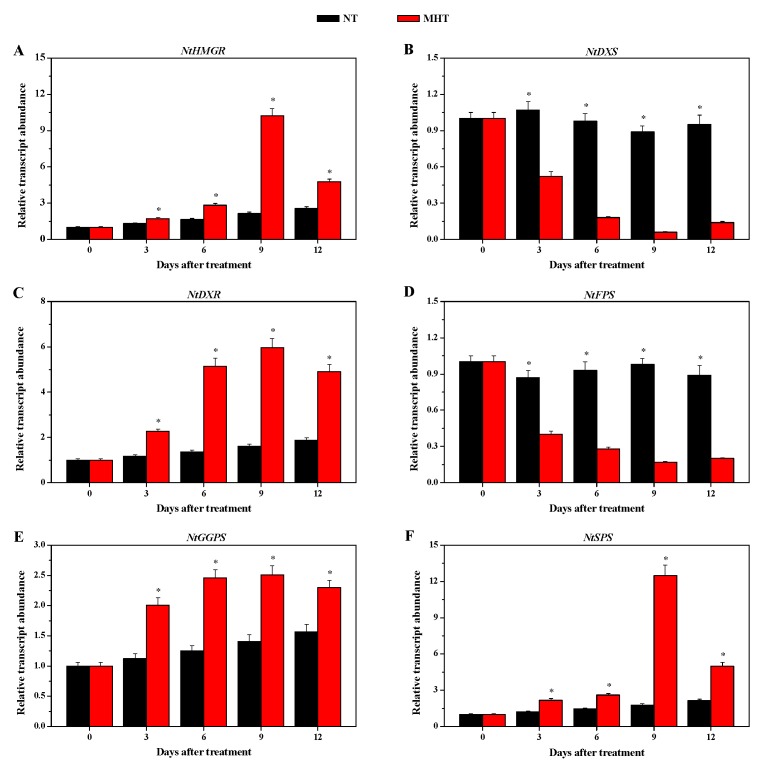
Relative transcript abundance of selected solanesol biosynthesis genes as measured by quantitative real-time PCR (qRT-PCR) using tobacco leaves grown at normal temperature (NT) and moderately high temperature (MHT). (**A**) *N. tabacum* 3-hydroxy-3-methylglutaryl-CoA reductase (*NtHMGR*), (**B**) *N. tabacum* 1-deoxy-d-xylulose 5-phosphate synthase (*NtDXS*), (**C**) *N. tabacum* 1-deoxy-d-xylulose 5-phosphate reductoisomerase (*NtDXR*), (**D**) *N. tabacum* farnesyl diphosphate synthase (*NtFPS*), (**E**) *N. tabacum* geranylgeranyl diphosphate synthase (*NtGGPS*), and (**F**) *N. tabacum* solanesyl diphosphate synthase (*NtSPS*). The asterisk (*) indicates *p* < 0.05.

The key enzymes in solanesol biosynthesis include DXS, DXR, farnesyl diphosphate synthase (FPS), geranylgeranyl diphosphate synthase (GGPS), and SPS [4,6]. 3-Hydroxy-3-methylglutaryl-CoA reductase (HMGR) catalyzes the conversion of HMG-CoA to mevalonate (MVA), which is the first step in the cytosolic pathway for isoprenoid biosynthesis in plants [38]. To understand how MHT affected solanesol biosynthesis, we monitored the relative transcript abundance of six key enzyme genes involved in solanesol biosynthesis, including two MVA (*NtHMGR* and *NtFPS*) and four MEP (*NtDXS*, *NtDXR*, *NtGGPS*, and *NtSPS*) genes, after MHT treatment. As expected, these genes were not markedly differentially expressed under NT and those under MHT treatment at 0 DAT (*p* > 0.05). However, at 3, 6, 9, and 12 DAT, the relative transcript abundance of *NtHMGR, NtDXR*, *NtGGPS*, and *NtSPS* was significantly higher, whereas that of *NtDXS* and *NtFPS* was significantly lower in MHT- than in NT-exposed tobacco leaves (*p* < 0.05) (Figure 5). 

## 4. Discussion

### 4.1. MHT Enhances the Solanesol Accumulation in Tobacco Leaves

As sessile organisms, plants adapt to rapidly changing environments by producing a diverse array of secondary metabolites, which play important roles in plant growth and development and in resistance to environmental stress, diseases, and pests [39]. Environmental factors, such as temperature, humidity, light intensity, the supply of water, minerals, and CO_2_, influence plant growth and secondary metabolite production [40]. In previous studies, high temperature (35 °C) treatment increased the hypericin, pseudohypericin, and hyperforin concentrations in the shoot tissues of St John’s wort (*Hypericum perforatum*) [41]. In the present study, compared to NT treatment, MHT treatment significantly increased the total solanesol content in tobacco leaves; MHT treatment for nine days led to a 1.661 times increase in leaf total solanesol content (Figure 1). Moreover, MHT treatment significantly increased the growth and photosynthesis in tobacco leaves (Figure 2), which can provide energy and carbohydrates for the solanesol biosynthesis in plants [6]. Similarly, the net photosynthetic rate and the Rubisco activity of tobacco leaves were higher at MHT (30 °C) than at NT (20 °C or 25 °C) [42], which promote the rapid biosynthesis of solanesol in tobacco under MHT treatment (Figure 1). Previous studies have shown that infection with TMV and *P. syringae* pv. *tabaci*, drought, shade, long-wavelength light, and rare earth element treatments all may cause an increase in solanesol content of tobacco leaves [2,13,16]. The present study indicates that temperature is an important environmental factor for the optimization of solanesol production in tobacco, and that controlled environment technology might facilitate the precise control of solanesol accumulation in tobacco leaves.

### 4.2. Transcriptome Analysis of Tobacco Leaves Treated with Moderately High Temperature

Through transcriptome analysis, we identified substantial differences in leaf gene expression between NT and MHT exposed plants. MHT treatment resulted in significant up- and downregulation of 492 and 1440 genes, respectively, as compared to NT (Figure 3). Mangelsen et al. [18] used the GeneChip microarray to investigate the response of developing barley (*Hordeum vulgare*) seeds after 0.5, 3, and 6 h high-temperature stress; 958 induced and 1122 repressed genes exhibited spatial and temporal expression patterns. Hancock et al. [17] investigated temperature-associated changes in gene expression in potato leaves and found that differences in functional categories under the elevated temperature regime included genes involved in secondary metabolism, lipid metabolism, hormone metabolism, photosynthesis, and amino acid biosynthesis. In the present study, functional enrichment analysis revealed that most of the DEGs were related to secondary metabolite biosynthesis, metabolic pathway, carbohydrate metabolism, lipid metabolism, hydrolase activity, catalytic activity, and oxidation-reduction process (Figure 4, Table 2). Moreover, 122 TFs of the DEGs were divided into 22 families, including MYB (16, 13.11%), AP2-EREBP (12, 9.84%), NAC (12, 9.84%), bHLH (10, 8.20%), and other TFs (72, 59.02%) (Table 3). 2514 tobacco TFs, including 274 AP2/ERF, 250 MYB, 190 bHLH, and 152 NAC tobacco TFs, have been reported in the study by Rushton et al. [43,44]. Furthermore, TFs also play an important role in regulating terpenoid (e.g., solanesol) biosynthesis, which largely determines the spatio-temporal expression of the enzyme genes and the specificity and efficiency of the induced expression [39]. Therefore, these candidate transcription factors identified in the present study will be helpful to study the molecular mechanisms of temperature regulation of solanesol accumulation.

### 4.3. Transcription of Solanesol Biosynthesis Genes in Tobacco Leaves Treated with Moderately High Temperature

Solanesol biosynthesis mainly involves two main stages: generation of the C5 isopentenyl diphosphate (IPP) precursor and its double-bond isomer dimethylallyl diphosphate (DMAPP), and generation of synthetic precursors (farnesyl diphosphate (FPP), geranylgeranyl diphosphate (GGPP), and solanesyl diphosphate (SPP)) [6]. Isopentenyl diphosphate and its isomer DMAPP are synthesized via two pathways that occur in different subcellular spaces; i.e., the MVA pathway located in the cytoplasm, and the MEP pathway in the plastids. These two pathways are not independent of each other, as IPP is shuttled between the cytoplasm and the plastids [45,46]. In the MVA pathway, HMGR catalyzes the conversion of HMG-CoA to MVA, which is the precursor of IPP [38,46]; FPS catalyzes the condensation of two IPP molecules with one DMAPP molecule to form one FPP molecule [6]. Schaller et al. [47] overexpressed the *Hevea brasiliensis HMGR* gene into the tobacco, resulting in an increase in HMGR activity and a six-fold increase in total sterol content. In the present study, MHT treatment led to a significant upregulation of the transcription level of *NtHMGR* in tobacco leaves, and a significant downregulation of the transcription level of *NtFPS* at 3, 6, 9, and 12 DAT (Figure 5). The presence of FPS was found in both cytoplasm and chloroplast of plants [48,49]. Notably, NtFPS has little effect on the rapid biosynthesis of solanesol in tobacco under MHT treatment. By contrast, as the first rate-limiting enzyme in the MVA metabolic pathway, NtHMGR likely plays a vital role in the rapid biosynthesis of solanesol in tobacco under MHT treatment.

DXS is the first enzyme of the MEP metabolic pathway that catalyzes the formation of 1-deoxy-d-xylulose 5-phosphate (DXP) from pyruvate and glycerol-3-phosphate, and DXR catalyzes the reduction of DXP to form MEP [5,6]. Two *N. tabacum DXR* genes, *NtDXR1* and *NtDXR2*, were identified by Zhang et al. [50]. Overexpression of *Synechosystis* sp. *DXR* genes in tobacco chloroplasts significantly increases the solanesol content in tobacco leaves [51]. Transient expression of potato *DXR* genes in *Nicotiana benthamiana* significantly increases the solanesol content [13]. In the present study, MHT treatment led to significant upregulation of the transcription level of *NtDXR* in tobacco leaves, and significant downregulation of the transcription level of *NtDXS* at 3, 6, 9, and 12 DAT (Figure 5). Notably, NtDXS has little effect on the rapid biosynthesis of solanesol in tobacco under MHT treatment. By contrast, NtDXR catalyzes the committed step of plastidial isoprenoid-precursor biosynthesis and plays a vital role in the rapid biosynthesis of solanesol in tobacco exposed to MHT.

In the MEP pathway, GGPS catalyzes the condensation of three IPP molecules with one DMAPP molecule to form one GGPP molecule; SPS catalyzes the synthesis of SPP from IPP, DMAPP, geranyl diphosphate (GPP), FPP, and GPPP [6,39]. Notably, SPP is the direct precursor of solanesol biosynthesis [2,5,6]. Transient expression of potato *GGPS* genes significantly increased the solanesol content of *N. benthamiana*, and co-expression of potato *SPS* genes with *DXS*, *DXR*, *IPI*, and *GGPS* genes significantly increased the solanesol content of *N. benthamiana* [13]. Overexpression of the tomato *SPS* gene in tobacco significantly increased the solanesol content in mature leaves [25]. In the present study, MHT treatment led to a significant upregulation of the transcription levels of *NtGGPS* and *NtSPS* in tobacco leaves at 3, 6, 9, and 12 DAT (Figure 5). Thus, as the key enzymes in the MEP pathway, NtGGPS and NtSPS play a vital role in the rapid biosynthesis of solanesol in tobacco under MHT treatment.

## 5. Conclusions

This study revealed that compared to growth at NT, MHT treatment results in a significant increase in solanesol content, dry weight, and net photosynthetic rate in tobacco leaves. In MHT-exposed tobacco leaves, 492 and 1440 genes were significantly up- and downregulated, respectively, as compared to plants exposed to NT. Functional enrichment analysis revealed that most of the DEGs were related to secondary metabolite biosynthesis, metabolic pathway, carbohydrate metabolism, lipid metabolism, hydrolase activity, catalytic activity, and oxidation-reduction process. Moreover, 122 TFs of the DEGs were divided into 22 families. The qRT-PCR results confirmed that MHT treatment led to a significant upregulation of the transcription levels of *NtHMGR*, *NtDXR*, *NtGGPS*, and *NtSPS* in tobacco leaves, and a significant downregulation of *NtDXS* and *NtFPS* transcription. This study indicates that solanesol accumulation in tobacco leaves can be manipulated through regulation of the environmental temperature and revealed the effect of MHT on the transcription of genes essential to solanesol biosynthesis, thus establishing a basis for further elucidation of the molecular mechanism of temperature regulation of solanesol accumulation. As far as we know, there are few studies on the TFs related to solanesol biosynthesis. Thus, future studies should gradually clarify the role of TFs in MHT induced solanesol biosynthesis.

## Figures and Tables

**Table 1:** Primers used for quantitative real-time PCR (qRT-PCR) in the present study

Gene Name	Forward Primer (5′–3′)	Reverse Primer (5′–3′)
*Actin*	CCACACAGGTGTGATGGTTG	GTGGCTAACACCATCACCAG
*NtHMGR*	GTCAGGTGGCGTGAGAAG	GTCCACGGCGGCTATCTT
*NtDXS*	ACCACCAACACCTCTTTT	TGATGACCAACATCCCAT
*NtDXR*	TGGTAAGAGGGTTCAGTGTT	CAGCCAGAGCATCTTTGAG
*NtFPS*	TGAGTTCCAGACTGCCTCT	GCCAATCTTACCCAGCAC
*NtGGPS*	CCCAATAAAACCTTCACTG	CACAGGTGGGTCTTTTACTA
*NtSPS*	GTTCCAGGTTGTTGATGAC	CTCGGAAAGGACTAGAAGG

**Table 2:** Eight statistically enriched gene ontology (GO) terms of DEGs between normal temperature (NT) and moderately high temperature (MHT) exposed tobacco leaves

GO ID	GO Terms	Type ^a^	Corrected *p*-Value	Test ^b^	Ref. ^c^
GO:0004553	Hydrolase activity, hydrolyzing *O*-glycosyl compounds	P	1.43 × 10^−9^	77	1039
GO:0016798	Hydrolase activity, acting on glycosyl bonds	P	1.43 × 10^−9^	80	1107
GO:0005975	Carbohydrate metabolic process	F	1.13 × 10^−7^	117	2085
GO:0006629	Lipid metabolic process	F	6.13 × 10^−7^	104	1831
GO:0005507	Copper ion binding	P	6.13 × 10^−7^	28	243
GO:0003824	Catalytic activity	P	6.13 × 10^−7^	810	23,582
GO:0055114	Oxidation-reduction process	F	2.26 × 10^−6^	199	4547
GO:0044710	Single-organism metabolic process	F	4.53 × 10^−6^	380	9926

^a^ GO ontology type: ‘F’ represents ‘biological process’, and ‘P’ represents ‘molecular function’. ^b^ Number of DEGs belonging to each GO term. ^c^ Total number of transcripts belonging to each GO term.

**Table 3:** Transcription factor (TF) analysis results of DEGs between normal temperature (NT) and moderately high temperature (MHT) exposed tobacco leaves

Gene Family	Number of TFs	Percentage (%)	TF Type
MYB	16	13.11	Transcription factor
AP2-EREBP	12	9.84	Transcription factor
NAC	12	9.84	Transcription factor
bHLH	10	8.20	Transcription factor
HB	9	7.38	Transcription factor
SBP	7	5.74	Transcription factor
C2C2-Dof	5	4.10	Transcription factor
LOB	5	4.10	Transcription factor
C3H	4	3.28	Transcription factor
CCAAT	4	3.28	Transcription factor
MADS	4	3.28	Transcription factor
Orphans	4	3.28	Transcriptional regulator
WRKY	4	3.28	Transcription factor
C2C2-GATA	3	2.46	Transcription factor
GRAS	3	2.46	Transcription factor
PLATZ	3	2.46	Transcription factor
bZIP	2	1.64	Transcription factor
C2H2	2	1.64	Transcription factor
LIM	2	1.64	Transcription factor
SNF2	2	1.64	Transcriptional regulator
TRAF	2	1.64	Transcriptional regulator
HSF	1	0.82	Transcription factor
LUG	1	0.82	Transcriptional regulator
S1Fa-like	1	0.82	Transcription factor
SET	1	0.82	Transcriptional regulator
TCP	1	0.82	Transcription factor
Trihelix	1	0.82	Transcription factor
zf-HD	1	0.82	Transcription factor

## Supplementary Materials

The following are available online at http://www.mdpi.com/2218-273X/8/4/165/s1, Table S1: Summary of sequencing results of transcriptome of tobacco leaves grown at normal temperature (NT) and moderately high temperature (MHT), Table S2: Sequence alignment results of reads generated from tobacco leaves grown at normal temperature (NT) and moderately high temperature (MHT) mapped to the reference genome, Table S3: The FPKM values of 122 TFs of DEGs between NT and MHT exposed tobacco leaves.
